# Biomarkers Associated with Immune-Related Adverse Events under Checkpoint Inhibitors in Metastatic Melanoma

**DOI:** 10.3390/cancers14020302

**Published:** 2022-01-08

**Authors:** Marcus Wölffer, Florian Battke, Martin Schulze, Magdalena Feldhahn, Lukas Flatz, Peter Martus, Andrea Forschner

**Affiliations:** 1 Department of Dermatology, University Hospital Tuebingen, 72076 Tuebingen, Germany; lukas.flatz@med.uni-tuebingen.de (L.F.); andrea.forschner@med.uni-tuebingen.de (A.F.); 2 Center for Genomics and Transcriptomics (CeGaT) GmbH, 72076 Tuebingen, Germany; florian.battke@cegat.de (F.B.); magdalena.feldhahn@cegat.de (M.F.); 3 Practice for Human Genetics, 72076 Tuebingen, Germany; martin.schulze@humangenetik-tuebingen.de; 4 Institute for Clinical Epidemiology and Applied Biostatistics (IKEaB), 72076 Tuebingen, Germany; peter.martus@med.uni-tuebingen.de

**Keywords:** immune-related adverse events, genetic alterations, immunotherapies, melanoma

## Abstract

**Simple Summary:**

Our aim was to check for possible associations between clinical parameters or NGS-based genetic alterations and the occurrence of immune-related adverse events (IRAE) in melanoma patients with immune checkpoint inhibitors (ICI). We analyzed 95 melanoma patients with ICI and were able to identify several biomarkers associated with the development of IRAE. Female sex was significantly associated with the development of hepatitis, increased total and relative monocytes at ICI initiation were significantly associated with the development of pancreatitis, the same, pre-existing autoimmune diseases. Furthermore, the following genetic alterations were identified being associated with IRAE: *SMAD3* (pancreatitis); *CD274*, *SLCO1B1* (hepatitis); *PRDM1*, *CD274* (encephalitis); *PRDM1*, *CD274*, *TSHR*, *FAN1* (myositis). Myositis and encephalitis, both, were associated with alterations of *PRDM1* and *CD274*, which might explain their joined appearance in clinical practice. Our findings can help to assess the risk for the development of IRAE in melanoma patients with ICI.

**Abstract:**

Immune checkpoint inhibitors (ICI) have revolutionized the therapeutic landscape of metastatic melanoma. However, ICI are often associated with immune-related adverse events (IRAE) such as colitis, hepatitis, pancreatitis, hypophysitis, pneumonitis, thyroiditis, exanthema, nephritis, myositis, encephalitis, or myocarditis. Biomarkers associated with the occurrence of IRAE would be desirable. In the literature, there is only little data available and furthermore mostly speculative, especially in view of genetic alterations. Our major aim was to check for possible associations between NGS-based genetic alterations and IRAE. We therefore analyzed 95 melanoma patients with ICI and evaluated their NGS results. We checked the data in view of potential associations between copy number variations (CNVs), small variations (VARs), human leucocyte antigen (HLA), sex, blood count parameters, pre-existing autoimmune diseases and the occurrence of IRAE. We conducted a literature research on genetic alterations hypothesized to be associated with the occurrence of IRAE. In total, we identified 39 genes that have been discussed as hypothetical biomarkers. We compared the list of these 39 genes with the tumor panel that our patients had received and focused our study on those 16 genes that were also included in the tumor panel used for NGS. Therefore, we focused our analyses on the following genes: *AIRE*, *TERT*, *SH2B3*, *LRRK2*, *IKZF1*, *SMAD3*, *JAK2*, *PRDM1*, *CTLA4*, *TSHR*, *FAN1*, *SLCO1B1*, *PDCD1*, *IL1RN*, *CD274*, *UNG*. We obtained relevant results: female sex was significantly associated with the development of hepatitis, combined immunotherapy with colitis, increased total and relative monocytes at therapy initiation were significantly associated with the development of pancreatitis, the same, pre-existing autoimmune diseases. Further significant associations were as follows: HLA homozygosity (hepatitis), and VARs on *SMAD3* (pancreatitis). Regarding CNVs, significant markers included *PRDM1* deletions and *IL1RN* (IRAE), *CD274* duplications and *SLCO1B1* (hepatitis), *PRDM1* and *CD274* (encephalitis), and *PRDM1*, *CD274*, *TSHR*, and *FAN1* (myositis). Myositis and encephalitis, both, were associated with alterations of *PRDM1* and *CD274*, which might explain their joined appearance in clinical practice. The association between HLA homozygosity and IRAE was clarified by finding HLA-A homozygosity as determining factor. We identified several genetic alterations hypothesized in the literature to be associated with the development of IRAE and found significant results concerning pre-existing autoimmune diseases and specific blood count parameters. Our findings can help to better understand the development of IRAE in melanoma patients. NGS might be a useful screening tool, however, our findings have yet to be confirmed in larger studies.

## 1. Introduction

In the past decade, immune checkpoint inhibitors (ICI) have revolutionized the therapeutic landscape of metastatic melanoma and significantly improved prognosis of metastatic melanoma patients [1]. ICI activate the endogenous immune response against tumor cells [1]. Anti-PD-1 inhibitors have also been approved by the EMA for adjuvant application after complete resection of metastases [2]. Combined ICI with ipilimumab and nivolumab is also successful in the neoadjuvant setting [3]. However, ICI might be associated with immune-related adverse events (IRAE), up to 59% in case of combined ICI [2]. Much research is being done in this area, yet up to now there are no genetically-based biomarkers associated with IRAE available. Since IRAE can be very limiting in the therapy management, especially in combined ICI, biomarkers associated with IRAE would be helpful, the more as IRAE can potentially be fatal [4].

Furthermore, the knowledge of biomarkers of distinct IRAE might help in the differential diagnosis, for example by distinguishing IRAE from laboratory abnormalities of other cause. Indeed, symptoms of IRAE might be unspecific in some cases, on the other side, early diagnosis is essential and may be life-saving [5].

A few biomarkers have already been identified, such as female sex [6], and pre-existing autoimmune diseases such as rheumatoid arthritis and inflammatory bowel disease [7]. Blood count parameters at occurrence of IRAE have been hypothesized to predict IRAE, including increased total leucocytes and relative neutrophil count, decreased relative lymphocyte count [8] and increased absolute and relative eosinophil counts [9]. However, they have not been systematically examined in larger studies yet and are not sufficient to be used in order to predict the occurrence of IRAE under ICI. Other markers put forward in literature concern specific genes involved in immune system regulation [10]. These include polymorphisms of *TSHR*, shown to affect the development of central tolerance and associated with autoimmune diseases [11,12], or polymorphisms of *PRDM1* found to influence antigen presentation [13] and associated with systemic lupus erythematodes [14]. Some authors describe in single case studies correlations between different HLA alleles and occurrence of IRAE [15,16]. Finally, existing literature does not satisfactorily differentiate between specific IRAE. In most of the cases, the authors refer to IRAE in general but not to the specific involved organs. However, markers specific for IRAE, especially for those with high fatality rates, would entail a great benefit.

In the following study, we sought to determine whether there are biomarkers associated with the occurrence of IRAE under ICI in melanoma patients. For this purpose, we evaluated NGS results and clinical data in view of the occurrence of IRAE in melanoma patients. We searched for the occurrence of IRAE in general as well as for specific IRAE and focused on germline genetic alterations known to play a central role in the regulation of the immune system as we hypothesized their influence also on the occurrence of IRAE.

## 2. Materials and Methods

We analyzed 95 melanoma patients that had been enrolled in a prospective study on the value of liquid biopsy and tumor sequencing between January 2018 and June 2018 who subsequently received immune checkpoint inhibitor (ICI) therapy. Details concerning tumor panel analysis and bioinformatics have already been reported [17]. The aim of this evaluation was to investigate a possible association between copy number variations (CNVs), small variations (VARs), human leucocyte antigen (HLA) and the occurrence of IRAE under ICI. After a thorough literature search (pubmed.ncbi.nlm.nih.gov, search terms “immunogenetics ipilimumab” and “genetics adverse events PD-1” on 27 January 2020), we found 39 genes that have been discussed in the literature. We compared the list of these 39 genes with the two different tumor panels that our patients had received for NGS by CeGat GmbH (see Table S2), including 711 and 742 genes respectively. Both panels are custom-design probe hybridization enrichment panels manufactured by Twist Bioscience, San Francisco, CA, USA. We found that 16 of the 39 genes were also included in the tumor panels used for NGS. We focused the evaluation on the following genes: *AIRE*, *TERT*, *SH2B3*, *LRRK2*, *IKZF1*, *SMAD3*, *JAK2*, *PRDM1*, *CTLA4*, *TSHR*, *FAN1*, *SLCO1B1*, *PDCD1 (PD1)*, *IL1RN*, *CD274 (PD-L1)*, *UNG*. Except for *AIRE*, both panels included all the genes mentioned, and *AIRE* was removed from the analysis due to lack of data. Therefore, we focused our analyses on these 16 genes. Our objective was also to check for possible associations between patient specific parameters such as sex, blood count, pre-existing autoimmune diseases and the occurrence of IRAE.

The patients either received combined immunotherapy or anti-PD-1 monotherapy. If they received both therapies in sequence, for example first anti-PD-1 monotherapy and later combined ICI, we focused on the data of the combined immunotherapy, as IRAE are more likely to occur here. Subsequently, we documented the time point of occurrence of IRAE, as well as the most common affected organs, in particular: colitis, pneumonitis, hepatitis, encephalitis, myocarditis, myositis, pancreatitis, exanthema, hypophysitis, nephritis, and thyroiditis. Regarding IRAE, we documented the date of first occurrence and the highest grade according to the Common Terminology Criteria of Adverse Events (CTCAE) scale from 1 to 5 [18], date of last administration of immunotherapy, median time to occurrence of specific IRAE, therapy and number of IRAE. We used the IRAE documentation of the oncologically experienced, treating physicians in the patient’s file. In case of late-occurring IRAE under follow-up therapies, IRAE were considered until 3 months after termination of ICI.

We then documented potential biomarkers for the occurrence of IRAE such as sex, type of immunotherapy, pre-existing autoimmune diseases, and blood count parameter at start of immunotherapy. Continuous variables, in particular blood count parameter, were divided into categories for better assessability (decreased, normal, increased, please refer to Table S1). Finally, we analyzed the results of the patients’ NGS results: VARs, CNVs, and HLA data. HLA-Class I genes were analyzed concerning heterozygosity and homozygosity, discerning HLA-A, HLA-B, and HLA-C homozygosity. We examined each selected gene for the effect of VARs. We abstained from further differentiating according to the exact variant location. We thus hypothesized that each polymorphism could lead to the same IRAE. The same procedure was done for CNVs affecting genes. We discerned deletions and duplications for IRAE in general, as well as colitis, hepatitis, and pancreatitis. We did not further differentiate between duplications and deletions for less common IRAE. CNVs above a certain frequency in the population are not recorded due to technical reasons, the difference to the comparison group not being large enough for the caller to respond. Consequently, it is possible that high-frequency CNVs are not considered in the analysis. All patients’ data were entered in and statistically analyzed with the statistical program for social sciences SPSS statistics version 25.0. (IBM Corp., Armonk, NY, USA), and Microsoft Excel Version 2019. The descriptive data was analyzed by absolute and relative frequency. We retained each variable that was in relative terms more frequently observed in patients with IRAE than in patients of the entire cohort. We proceeded correspondingly with the three most prevalent IRAE for each potential marker. We obtained the potentially significant biomarkers for occurrence of IRAE and proceeded by testing these markers for significance. The exact version of the Chi-Squared-Test was used for statistical significance. The level of significance was set at 0.05 in all analyses. Results of organ specific IRAEs and biomarkers with a prevalence outside the interval of 33% to 66% are purely exploratory. This is due to the limited sample size of our work and the number of biomarkers to be detected, especially in the Section 3.6 and Section 3.7. Within this restriction, differences in proportions of at least 30% could be detected confirmatory with a power of at least 80% (Chi-square test for unequal samples with ratio at most 2:1, *n* = 95 subjects). Our results support the markers previously suggested in the literature but need to be confirmed and further specified in future more comprehensive studies as correction for multiple testing was not feasible due to lack of power.

All subjects gave their informed consent for the inclusion and participation in the study. The study was conducted in accordance with the Declaration of Helsinki, and the protocol was approved by the Ethics Committee of the Ärztekammer Baden-Württemberg, and the local Ethics Committee of the Eberhard-Karls-University Tübingen, approval numbers F-2016-010 and 827/2018BO2.

## 3. Results

Our results must be interpreted with caution due to the exploratory approach. Table 1 and Table 2 show clinical and genetic characteristics of the cohort.

### 3.1. Sex

A total of 24.4% of female patients developed hepatitis (*p* = 0.021), compared to only 7.4% of male patients, whereas 18.5% of male patients developed pancreatitis, opposed to only 7.3% of female patients.

### 3.2. Type of Therapy

Combined immunotherapy led to more IRAE than monotherapy. 67.8% of patients with combined therapy developed IRAE, compared to 52.8% of patients with monotherapy. 30.5% of patients with combined therapy compared to 5.6% with monotherapy (*p* = 0.004) developed colitis. 18.6% of patients with combined therapy had hepatitis, compared to 8.3% of patients with monotherapy. Finally, 16.9% of patients with combined therapy had pancreatitis, compared to 8.3% of patients with monotherapy.

### 3.3. Laboratory Data

Elevated levels of protein S100 or lactate dehydrogenase (LDH) at therapy initiation were not associated with IRAE.

In the differential blood cell count at therapy initiation, increased leucocytes were associated with colitis, hepatitis or pancreatitis. Increased total neutrophils were associated with colitis or pancreatitis. Decreased relative lymphocytes were associated with pancreatitis. Increased total and relative monocytes were also associated with IRAE. Increased total monocytes were associated with colitis, hepatitis, or pancreatitis (*p* < 0.0005), whereas increased relative monocytes were only associated with colitis or pancreatitis (*p* = 0.001). The single patient having had high levels of eosinophils at start of immunotherapy developed colitis, pancreatitis and nephritis during immunotherapy. Table 3 shows laboratory results and frequency of IRAE, colitis, hepatitis or pancreatitis.

### 3.4. Pre-Existing Autoimmune Diseases

Pre-existing autoimmune diseases were associated with the occurrence of IRAE, 6 out of 8 patients with pre-existing autoimmune diseases developed IRAE. A total of 100% of 4 patients suffering from rheumatoid arthritis, vitiligo, or Crohn’s disease developed IRAE. Table 4 shows IRAE occurrence in patients with different pre-existing autoimmune diseases.

The occurrence of colitis was associated with rheumatoid arthritis (50%), Crohn’s disease (100%), or heparin-induced thrombopenia type II (HIT-II; 100%). The development of hepatitis was associated with diabetes mellitus Type 1 (50%), vitiligo (100%), or HIT-II (100%). The development of pancreatitis was associated with rheumatoid arthritis (50%), vitiligo (100%), or HIT-II (100%). Pancreatitis was linked significantly to pre-existing autoimmune diseases (*p* = 0.041).

### 3.5. HLA

A total of 74% of HLA homozygous patients developed IRAE, compared to 58% of HLA heterozygous patients. This difference was not statistically significant. We differentiated further between HLA-A, -B, and -C homozygosity. A total of 77% of HLA-A homozygous patients developed IRAE, compared to only 60% of HLA-A heterozygous patients. Notably, only 50% of HLA-B and 63% of HLA-C homozygous patients developed IRAE, compared to 63% and 62% of HLA-B and HLA-C heterozygous patients, respectively. HLA-A homozygosity thus seems to be the determining factor in HLA homozygosity-associated IRAE.

A positive correlation between IRAE and homozygosity was found for HLA-A and HLA-C homozygosity for colitis, and HLA-A, HLA-B, and HLA-C homozygosity for hepatitis. HLA homozygosity was significantly linked to the development of hepatitis (*p* = 0.015). HLA-B homozygosity is seemingly a protective factor in general IRAE, colitis or pancreatitis. Table 5 illustrates the results on HLA homo- and heterozygosity.

We analyzed confidence intervals and odds ratio for HLA homo- and heterozygosity for IRAE, colitis, hepatitis and pancreatitis (see Figure S1).

### 3.6. VARs

VARs on *SMAD3* were significantly linked to the development of pancreatitis (*p* = 0.034). This result must be read and interpreted with caution due to the exploratory approach. Table 6 shows genes affected by VARs, which were associated with the development of IRAE, for IRAE in general and each IRAE in particular.

### 3.7. CNVs

*IKZF1*, *FAN1* and *IL1RN* were linked to IRAE in general, as well as all three main IRAE in particular. Deletions were found to be more frequently associated with IRAE than duplications. CNVs on *IL1RN* (*p* = 0.033) and deletions on *PRDM1* (*p* = 0.026) were significantly linked to IRAE. Duplications on *CD274* (*p* = 0.043) and CNVs on *SLCO1B1* (*p* = 0.010) were significantly linked to hepatitis. These results must be interpreted with caution due to the exploratory approach. We distinguished deletions and duplications for each CNV for IRAE in general, as well as colitis, hepatitis and pancreatitis (see Table 7).

We analyzed confidence intervals and odds ratio for CNVs for IRAE, colitis, hepatitis and pancreatitis (see Figure S2). CNVs on CTLA-4 were eliminated from the forest plot for pancreatitis due to lack of data.

CNVs on *PRDM1* and *CD274* were significantly linked to encephalitis (*p* = 0.014 and *p* = 0.032) and myositis (*p* = 0.014 and *p* = 0.032). CNVs on *TSHR* and *FAN1* were significantly linked to myositis (*p* = 0.049 and *p* = 0.039). These results must be interpreted with caution due to the exploratory approach. Since a distinction between duplications and deletions would have made the sample size too small for statistical statements for less common specific IRAE, this has been omitted here (see Table 8).

## 4. Discussion

We found a significant link between female sex and hepatitis, and a correlation between male sex and pancreatitis. Combined immunotherapy was associated with a higher incidence of IRAE and significantly linked to colitis. We found an association between increased leucocytes at start of immunotherapy and occurrence of colitis, hepatitis or pancreatitis. Increased absolute neutrophils at start of immunotherapy were associated with colitis or pancreatitis, whereas decreased relative lymphocytes at start of immunotherapy were associated with pancreatitis. Increased total and relative monocytes at start of immunotherapy were associated with IRAE or colitis. Increased absolute and relative monocytes at start of immunotherapy were significantly linked to the occurrence of pancreatitis. We found furthermore a significant link between pre-existing autoimmune diseases and pancreatitis. HLA homozygosity was linked to IRAE in general or colitis. HLA homozygosity was significantly associated with hepatitis. HLA-A homozygosity was strikingly linked to the occurrence of IRAE in general, colitis or hepatitis. VARs on *SMAD3* were significantly linked to pancreatitis. CNVs on *IKZF1*, *FAN1* and *IL1RN* were linked to IRAE, colitis, hepatitis or pancreatitis. CNVs on *IL1RN* and deletions on *PRDM1* were significantly linked to IRAE, whereas duplications on *CD274* and CNVs on *SLCO1B1* were significantly linked to hepatitis. Finally, CNVs on *PRDM1* and *CD274* were significantly linked to encephalitis, and CNVs on *PRDM1*, *CD274*, *TSHR* and *FAN1* were significantly linked to myositis.

If we now look in the literature to see how our results fit with what has already been published, we find that in terms of sex differences, female sex is a known risk factor for the development of autoimmune diseases, especially autoimmune hepatic diseases [19], and is linked to the occurrence of IRAE during Anti-CTLA-4 monotherapy [6]. Thus, our results fit very well to what is known. Furthermore, Kitagataya et al. [20] reported an association between female sex and autoimmune hepatitis under immunotherapy. However, the underlying pathomechanism is not fully understood yet. Unlike hepatitis, pancreatitis was more common in the male sex, which also fits very well with the literature. Others also reported an association between male sex and pancreatic injury during immunotherapy [21]. Additional information is available on type I autoimmune pancreatitis (IgG4-related pancreatitis), which has also been shown to be linked to male sex [22]. In addition, male patients had worse responses to glucocorticoid therapy, more relapses of pancreatitis and higher levels of peripheral eosinophil count [23], the latter also being linked to IRAE [9]. Recent studies showed a significant correlation between western diet and autoimmune pancreatitis in mice, whereas caloric restriction halved the occurrence [24]. Furthermore, chronic pancreatitis was detected more frequently in men, and alcohol has been shown to be its most important risk factor [25]. Therefore, male patients might be more likely to develop pancreatic IRAE because of their diet.

Concerning the type of ICI, in the CheckMate 067 study there was a considerably higher incidence of IRAE during combined immunotherapy compared to Anti-PD-1 monotherapy [26]. This fits very well to the findings of our study. We also noted a comparable impact of type of immunotherapy on frequency of IRAE in our cohort. Recently, it has also been shown that dosage of ipilimumab and nivolumab in combined immunotherapy seems to be decisive for the occurrence of IRAE. Lebbé et al. showed that nivolumab 3 mg/kg combined with ipilimumab 1 mg/kg as opposed to the established dosage Nivolumab 1 mg/kg and ipilimumab 3 g/kg was associated with a significantly lower occurrence of grade 3 to 5 IRAE while survival outcomes and treatment response were similar between the two dosages [27]. This dosage has also been identified as the optimal dosage for combined immunotherapy in a neoadjuvant setting as it presented comparable response rates and lower incidence of IRAE during the opACIN-neo trial [28].

Furthermore, changes in blood cell counts during immunotherapy had been found to be predictive for the risk for IRAE. These laboratory findings included increased total leucocytes and relative neutrophil count, and decreased relative lymphocyte count [8], as well as increased absolute and relative eosinophil counts for endocrinological IRAE [9]. Corresponding to these reports, we also found an association between increased leucocytes at start of immunotherapy and the occurrence of IRAE, such as colitis, hepatitis or pancreatitis. We also found an association between increased absolute number of neutrophils at start of immunotherapy and the occurrence of colitis or pancreatitis. Finally, decreased relative number of lymphocytes at the start of immunotherapy was linked to pancreatitis. However, these results were not statistically significant. It has to be considered, that Fujisawa et al. analyzed blood counts at the start of IRAE [8], whereas we considered blood count data already at start of immunotherapy. Given the parallels in our results, we assume that these markers might be indicative for IRAE. We could not confirm the reported correlation between higher total and relative levels of eosinophils and the occurrence of IRAE [9], probably due to the limited sample size of our cohort. We had only one single patient in our cohort with high levels of eosinophils at start of immunotherapy. This patient indeed developed colitis, pancreatitis and nephritis during immunotherapy.

We found furthermore an association between increased absolute and relative monocytes at the start of immunotherapy and the occurrence of IRAE or colitis. Increased total and relative monocytes were significantly associated with pancreatitis. Increased monocyte count has been linked to decreased overall survival in melanoma patients [29]. This would suggest a correlation of increased monocyte count with both decreased overall survival and occurrence of IRAE, which contradicts findings in recent literature about a supposed link between response and occurrence of IRAE [30]. A history of autoimmune disease has been shown to be associated with a higher incidence of IRAE during immunotherapy of melanoma [31]. Several studies analyzed risk of exacerbation and occurrence of IRAE depending on the type of therapy. Johnson et al. noted exacerbations of rheumatoid arthritis and inflammatory bowel disease (IBD), as well as increased occurrence of IRAE, during anti-CTLA-4 monotherapy [7]. Menzies et al. showed that melanoma patients with pre-existing autoimmune diseases had a risk of exacerbation when administered anti-PD-1 therapy, but no increased risk of developing de novo IRAE compared to general population [32]. The inhibition of CTLA-4 or PD-1/PD-L1 pathways in the gastrointestinal tract has been shown to intensify the immune response. Moreover, patients with pre-existing IBD in particular have been shown to be predisposed to an exacerbation of IBD/development of colitis under ICI [33]. Meserve et al. [34] showed that 40% of IBD patients experienced flares during ICI, often requiring corticosteroids (76%) or biologicals (37%). However, these flares were mostly manageable, and only rarely led to therapy discontinuation (35%) [34]. The same, Abdel-Wahab et al. have shown that IRAE occurring in patients with pre-existing autoimmune diseases only rarely led to termination of therapy. Nevertheless, considering that patients with autoimmune diseases developing exacerbations or IRAE have response rates at least as high as in the general population [35], they should not be excluded from therapy.

Although there is a substantial lack of information in the literature about links between HLA and IRAE, several findings point out that there might be an association between specific HLA-A alleles and HLA homozygosity on one hand, and response to immunotherapy, autoimmune diseases, as well as IRAE occurrence on the other.

Li et al. noted in a case report that an HLA-A*02:01 homozygous patient with metastatic lung squamous cell cancer treated with nivolumab showed durable remission after occurrence of severe immune-related pneumonitis [15]. Hayashi et al. [36] found the HLA-A downstream regulatory region to be the decisive factor in pathogenesis of autoimmune vitiligo. Through increase of HLA-A expression and HLA-A*02:01 protein, which presents several vitiligo autoimmune antigens, it facilitates recognition and attack of melanocytes by autoreactive T cells [36]. We noted an association between HLA-I homozygosity and the occurrence of IRAE, colitis or hepatitis. HLA-A homozygosity was strikingly associated with IRAE, colitis, or hepatitis, proving to be the determining factor of this association. To our best knowledge, this finding has not yet been identified in literature.

Other authors concluded that HLA-class I homozygosity might be linked to inferior outcome and found simultaneous heterozygosity on all HLA-I loci (A, B and C) to be associated with higher survival rates compared to patients homozygous on at least one HLA-I locus [37]. Inferior outcome was explained by elements impairing T-cells’ recognition of tumor antigens on HLA-B. This coincides with our results about HLA-B homozygosity being a protective factor for the occurrence of IRAE, when considering recent results about an association between IRAE occurrence and treatment response [30].

It seems imperative to differentiate between HLA-A, HLA-B, and HLA-C homozygosity as they seem to have opposite effects on treatment response and IRAE occurrence.

In our study, we found out that VARs on *SMAD3* were significantly linked to the development of pancreatitis: 33% of patients with *SMAD3* variants suffered from pancreatitis while the incidence of pancreatitis in the complete cohort was only 14%. To our best knowledge, *SMAD3* has not yet been linked to IRAE. Of note, *SMAD3* is known as a key molecule in TGF-β signaling pathway and studies have shown the inhibitory effect of *SMAD3* and the TGF-β pathway on natural killer cells in the tumor microenvironment: disruption of *SMAD3* in natural killer cells was associated with enhanced activity of natural killer cells and cytokine production [38]. If *SMAD3* plays an immunosuppressive role, as shown by Wang et al., an affection of *SMAD3* by VARs could lead to a dysfunction potentially explaining an IRAE. We observed a correlation between *SMAD3* affected by VARs and partial response at first staging, which confirms findings about the association between response and occurrence of IRAE [30].

Concerning the CNVs, it is interesting, that higher numbers of CNVs in melanoma tissue have been linked to a higher incidence of metastases, recurrence and death [39]. In the literature [10] we furthermore found an association between different genetic loci and IRAE which we strived to confirm. It has also been reported that responsiveness to ICI might be linked to specific polymorphisms [40], including rs419598 on *IL1RN*. The same, a higher incidence of toxicity under ICI was found being associated with alterations of *CD274*.

CNVs on *IL1RN* were significantly linked to IRAE in general and associated with all three main IRAE in particular, i.e., colitis, hepatitis and pancreatitis. If the role of CNVs on *IL1RN* concerning the occurrence of IRAE during immunotherapy is not yet fully understood, *IL1RN* is known for its role in the development of autoimmune diseases. Yoshizaki et al. showed an increased risk for the development of autoimmune aortitis in *IL1RN*-deficient mice, which they explained by stronger signaling of IL-1 [41]. Polymorphisms on *IL1RN* have been found to be associated with the occurrence of Hashimoto’s thyroiditis and to predict severity [42].

CNVs on *CD274* (PD-L1) were a recurrent marker for IRAE, being significantly linked to hepatitis, encephalitis and myositis. Once again, the role of CNVs on *CD274* in view of the occurrence of IRAE during immunotherapy has not yet been fully understood. Polymorphisms on *CD274* are however known to be associated with multiple autoimmune diseases, such as type 1 diabetes [43], ankylosing spondylitis [44], and Graves’ disease as well as autoimmune Addison’s disease [45].

Polymorphisms on *PRDM1* have been associated with systemic lupus erythematodes (SLE) [14]. More precisely, rs548234 has been found to be a risk allele on *PRDM1* associated with SLE, decreasing the expression of BLIMP1 (B-lymphocyte-induced maturation proteine-1) in dendritic cells, which is involved in immunological tasks such as antigen presentation [13]. Polymorphisms on *PRDM1* have also been associated with other autoimmune diseases such as rheumatoid arthritis and inflammatory bowel disease [46]. The role of *PRDM1* in preventing autoimmunity has been analyzed in mice by Roberts et al. [47]. The authors showed that *PRDM1* is expressed by medullary thymic epithelial cells, involved in the deletion of self-reactive T cells and the development of regulatory T cells. Deletion of *PRDM1* resulted in autoimmune pathology [47]. Our results confirm these findings: deletions of *PRDM1* resulted in IRAE. Xia et al. revealed that homozygous deletions of *PRDM1* correlated with decreased plasma cell differentiation and upregulation of genes involved in tumor cell proliferation in activated B-cell-like diffuse large B-cell lymphoma (ABC-DLBCL) patients [48]. Xia et al. also found that patients with *PRDM1* deletions were showing upregulation of transcription factors such as STAT3. STAT3 has been identified as main factor in increased PD-L1 expression on tumor cells [49], which is targeted by anti-PD-1 therapy. *PRDM1* deletions may thus be regarded as factor supporting Anti-PD-1 therapy, and thereby inducing IRAE. Of note, the association of CNVs on *PRDM1* to encephalitis and myositis has not been reported in the literature yet. It would certainly be interesting if this could be tested in larger cohorts.

Polymorphisms of *TSHR* in enhancer regions, especially rs4411444 and rs4903961, are known to be associated with autoimmune diseases such as Graves’ disease and Hashimoto’s disease [11]. Polymorphisms of *TSHR* have been shown to affect the development of central tolerance, thus explaining the occurrence of autoimmune phenomena such as Graves’ disease [12].

Polymorphisms of *SLCO1B1* have not yet been found to be associated with autoimmune diseases or IRAE. *SLCO1B1* is a drug transporter and member of the solute carrier family, and polymorphisms of *SLCO1B1* have been associated with drug metabolism disorders such as atorvastatin-induced adverse events [50] or sorafenib-induced adverse events [51]. Polymorphisms of *SLCO1B1* have also been associated with the occurrence of methimazole-induced liver injury in patients with Grave’s disease [52].

*FAN1* has not yet been associated with autoimmune diseases or IRAE. *FAN1* plays an important role in the removal of DNA interstrand crosslinks [53], and has been identified as a protective factor in the occurrence and progression of Huntington’s disease [54].

The occurrence of myositis as well as encephalitis was associated with CNVs on *CD274* and *PRDM1* in our study, possibly explaining the combined occurrence of these IRAE in clinical practice. Sato et al. also reported the frequent combined occurrence of neurological IRAE such as myasthenia gravis, encephalitis and meningitis with myositis during immunotherapy [55]. Reynolds et al. confirmed these findings, showing that neurological IRAE often overlap between one another and are associated with myositis [56].

Although it is currently assumed that interruption or even termination of ICI due to severe side effects has no impact on the prognosis, i.e., the survival of patients [57], the development of severe side effects can be very disabling, even life-threatening for patients.

Our study can help to estimate the risk of patients to develop IRAE.

In summary, to estimate the risk of developing IRAE, on the one hand, we have markers such as gender, blood count parameters, and pre-existing autoimmune diseases, which are easy to obtain; on the other hand, NGS-based results are a more complex and expensive option. However, NGS provides us with additional information and should be considered in risk assessment, especially when multiple therapies are available.

Of course there are several limitations in our study. First, results of organ-specific IRAEs and biomarkers with a prevalence outside the interval of 33% to 66% are purely exploratory. This is due to the limited sample size of our work and the number of biomarkers to be detected, especially in the Section 3.6 and Section 3.7. Our results may well support the biomarkers previously described in the literature but will have to be confirmed in a larger sample size. Second, we did not differentiate between CNVs duplications and deletions for our analysis of less prevalent IRAE, as our sample size was too small to perform further differentiation. Moreover, CNV detection from NGS data on single exon level is less accurate compared to methods such as qPCR or MLPA. Thus, confirmation of identified CNV markers by an alternative method in a larger cohort seems advisable to avoid misinterpretations due to technical limitations. We furthermore did not distinguish between individual VARs and simplified that most of them were similarly located on each gene, and had thereby the same effect.

However, there is also great strength in our study. We have carefully evaluated the patient records and can therefore assume with a high degree of confidence that the clinical data are accurate. We used a comprehensive tumor panel, which increases the chance of identifying biomarkers for IRAE. By specifically differentiating distinct IRAE, HLA-I homozygosity, CNVs deletions and duplications, we have been able to obtain important results. 

## 5. Conclusions

Our study can help to define biomarkers associated with the occurrence of IRAE in general and of several specific IRAE. We found a significant association between several genetic markers and the occurrence of IRAE, which were merely hypothesized in the literature. We propose that in future, basic screening for biomarkers associated with the occurrence of IRAE should be carried out before initiation of ICI, in particular in patients for whom a therapeutic alternative, for example with BRAF-/MEK inhibitors is possible, even though ICI is a mainstay of therapy for BRAF-mutated patients as well [58]. Where NGS data is already available, a focused query should be made regarding the presence of potentially relevant polymorphisms in genes associated with the development of IRAE. If future studies support our findings by validation or potentially the discovery of additional genetic biomarkers, NGS may become the screening method of choice.

As ICI are used more and more frequently across many different cancer types, further studies on biomarkers associated with the development of IRAE should be performed.

## Figures and Tables

**Table 1:** Clinical characteristics of the cohort.

Clinical Characteristics	Monotherapy with Anti-PD-1 Antibody		Combined with Ipilimumab and Nivolumab		Total	
	Number of Patients	%	Number of Patients	%	Number of Patients	%
Total	36	100	59	100	95	100
Sex						
Male	18	50	36	61	54	57
Female	18	50	23	39	41	43
Melanoma type						
Cutaneous	27	75	36	61	63	66
Acral lentiginous	3	8	6	10	9	9
Mucosal	0	0	3	5	3	3
Uveal	2	6	4	7	6	6
Occult	3	8	9	15	12	13
Other	1	3	1	2	2	2
AJCC cancer stage at study inclusion						
III	18	50	2	3	20	21
IV	18	50	57	97	75	79
First line						
Yes	32	89	26	44	58	61
No	4	11	33	56	37	39
Intention						
Adjuvant	20	56	0	0	20	21
Palliative	16	44	59	100	75	79
Origin of tissue sequenced						
Lymph node metastasis	13	36	18	31	31	33
Other metastasis	15	42	26	44	41	43
Primary melanoma	6	17	9	15	15	16
CNS metastasis	0	0	1	2	1	1
Local recurrence	0	0	1	2	1	1
Unknown	2	6	4	7	6	6
BRAF mutation						
Positive	14	39	31	53	45	47
Negative	22	61	28	47	50	53
TMB values at start immunotherapy						
Low (>3.3 Var/Mb)	8	22	21	36	29	31
Intermediate (3.3–23.1 Var/Mb)	19	53	25	42	44	46
High (>23.1 Var/Mb)	9	25	10	17	19	20
Not determinable	0	0	3	5	3	3
Pre-existing autoimmune diseases						
Diabetes Mellitus Type 1	0	0	2	3	2	2
Rheumatoid Arthritis	1	3	1	2	2	2
Vitiligo	0	0	1	2	1	1
Crohn’s Disease	0	0	1	2	1	1
Other	0	0	2	3	2	2
IRAE under immunotherapy						
Occurrence of IRAE	19	53	40	68	59	62
Colitis	2	6	18	31	20	21
Hepatitis	3	8	11	19	14	15
Pancreatitis	3	8	10	17	13	14
Hypophysitis	1	3	9	15	10	11
Pneumonitis	2	6	6	10	8	8
Thyroiditis	1	3	6	10	7	7
Exanthema	1	3	6	10	7	7
Nephritis	1	3	2	3	3	3
Myositis	0	0	3	5	3	3
Encephalitis	1	3	2	3	3	3
Myocarditis	1	3	1	2	2	2
Protein S100 at start of immunotherapy						
S100 elevated	8	22	36	61	44	46
S100 normal	28	78	23	39	51	54
LDH at start of immunotherapy						
LDH elevated	5	14	26	44	31	33
LDH normal	31	86	33	56	64	67
Differential blood count at start of immunotherapy						
Leucocytes						
Normal	34	94	46	78	80	84
Increased	2	6	8	14	10	11
Decreased	0	0	5	8	5	5
Neutrophils abs.						
Normal	32	89	46	78	78	82
Increased	4	11	9	15	13	14
Decreased	0	0	4	7	4	4
Neutrophils%						
Normal	34	94	51	86	85	89
Increased	1	3	6	10	7	7
Decreased	1	3	2	3	3	3
Lymphocytes abs.						
Normal	24	67	47	80	71	75
Increased	1	3	1	2	2	2
Decreased	11	31	11	19	22	23
Lymphocytes %						
Normal	17	47	36	61	53	56
Increased	2	6	2	3	4	4
Decreased	17	47	21	36	38	40
Monocytes abs.						
Normal	32	89	49	83	81	85
Increased	3	8	9	15	12	13
Decreased	1	3	1	2	2	2
Monocytes %						
Normal	34	94	54	92	88	93
Increased	2	6	5	8	7	7
Decreased	0	0	0	0	0	0
Eosinophils abs.						
Normal	31	86	49	83	80	84
Increased	1	3	0	0	1	1
Decreased	4	11	10	17	14	15
Eosinophils %						
Normal	31	86	46	78	77	81
Increased	1	3	0	0	1	1
Decreased	4	11	13	22	17	18

AJCC: American Joint Committee on Cancer; CNS: Central Nervous System; BRAF: B-Rapidly Accelerated Fibrosarcoma; TMB: Tumor Mutational Burden; IRAE: Immune-Related Adverse Event; LDH: Lactate Dehydrogenase.

**Table 2:** Genetic characteristics of the cohort.

Genetic Characteristics	No. Patients	%
HLA		
Homozygosity	23	24
Heterozygosity	72	76
HLA-A		
Homozygosity	13	14
Heterozygosity	82	86
HLA-B		
Homozygosity	8	8
Heterozygosity	87	92
HLA-C		
Homozygosity	8	8
Heterozygosity	87	92
Small sequence variations (VARs)		
*AIRE*	1	1
*TERT*	7	7
*SH2B3*	66	70
*LRRK2*	95	100
*IKZF1*	0	0
*SMAD3*	12	13
*JAK2*	3	3
*PRDM1*	45	47
*CTLA4*	59	62
*TSHR*	95	100
*FAN1*	62	65
*SLCO1B1*	57	60
*PDCD1*	3	3
*IL1RN*	48	50
*CD274*	4	4
*UNG*	2	2
Copy number variations (CNVs)		
*AIRE*	1	1
Duplications	0	0
Deletions	1	1
Both	0	0
*TERT*	45	47
Duplications	37	39
Deletions	7	7
Both	1	1
*SH2B3*	27	28
Duplications	26	27
Deletions	1	0
Both	0	0
*LRRK2*	38	40
Duplications	27	28
Deletions	10	11
Both	1	1
*IKZF1*	40	42
Duplications	34	36
Deletions	4	4
Both	2	2
*SMAD3*	36	38
Duplications	36	38
Deletions	0	0
Both	0	0
*JAK2*	43	45
Duplications	20	21
Deletions	23	24
Both	0	0
*PRDM1*	32	34
Duplications	10	11
Deletions	22	23
Both	0	0
*CTLA4*	14	15
Duplications	9	9
Deletions	4	4
Both	1	1
*TSHR*	20	21
Duplications	7	7
Deletions	10	11
Both	3	3
*FAN1*	40	42
Duplications	25	26
Deletions	11	12
Both	4	4
*SLCO1B1*	59	62
Duplications	41	43
Deletions	11	12
Both	7	7
*PDCD1*	28	29
Duplications	24	25
Deletions	4	4
Both	0	0
*IL1RN*	15	16
Duplications	9	9
Deletions	5	5
Both	1	1
*CD274*	18	19
Duplications	3	3
Deletions	15	16
Both	0	0
*UNG*	31	33
Duplications	27	28
Deletions	3	3
Both	1	1

HLA: Human Leukocyte Antigen.

**Table 3:** Distribution of laboratory results in our cohort in relation to development of IRAE in percent.

Laboratory Results			*N*	IRAE	Colitis	Hepatitis	Pancreatitis
			(95)	%	%	%	%
Leucocytes		Normal Increased	80 10	61 60	18 **40**	13 **30**	11 **30**
		Decreased	5	80	40	20	20
Neutrophils	Absolute	Normal Increased	78 13	62 62	18 **39**	14 15	10 **31**
		Decreased	4	75	25	25	25
	%	Normal Increased	85 7	66 29	21 29	15 14	14 14
		Decreased	3	33	0	0	0
Lymphocytes	Absolute	Normal Increased	71 2	66 50	24 0	17 0	14 0
		Decreased	22	50	14	9	14
	%	Normal Increased	53 4	70 25	25 0	21 0	11 0
		Decreased	38	55	18	8	**18**
Monocytes	Absolute	Normal Increased	81 12	62 **75**	20 **33**	14 **25**	9 **50** (*p* < 0.0005)
		Decreased	2	0	0	0	0
	%	Normal Increased	88 7	61 **71**	19 **43**	16 0	10 **57** (*p* = 0.001)
		Decreased	0	-	-	-	-
Eosinophils	Absolute	Normal Increased	80 1	68 100	21 100	16 0	14 100
		Decreased	14	29	14	7	7
	%	Normal Increased	77 1	68 100	21 100	17 0	14 100
		Decreased	17	35	18	6	6

Results analyzed in the main text are highlighted in bold.

**Table 4:** IRAE occurrence in patients with pre-existing autoimmune disease.

Pre-Existing Autoimmune Disease	N (% Occurred IRAE)	IRAE Occurred
Diabetes mellitus type I	2 (50%)	Hepatitis, pneumonitis
Rheumatoid arthritis	2 (100%)	Pancreatitis, colitis
Vitiligo	1 (100%)	Exanthema, hepatitis, pancreatitis
Crohn’s disease	1 (100%	Colitis
HIT type II	1 (100%)	Colitis, thyroiditis, hepatitis, pancreatitis, nephritis
AIHA	1 (0%)	-

**Table 5:** Distribution of HLA zygosity in our cohort, subset by HLA class I locus in relation to development of IRAE in absolute values and percent.

HLA Class I locus	Patients Total		IRAE		Colitis		Hepatitis		Pancreatitis	
	(N)	(%)	(N)	(%)	(N)	(%)	(N)	(%)	(N)	(%)
Total	95	100	59	62	20	21.05	14	14.74	13	13.68
HLA Homozygosity Heterozygosity	23 72	24 76	17 42	**74** 58	8 12	35 17	7 7	**30** (*p* = 0.015) 10	3 10	13 14
HLA-A Homozygosity Heterozygosity	13 82	14 86	10 49	**77** 60	5 15	**38** 18	4 10	**31** 12	2 11	15 13
HLA-B Homozygosity Heterozygosity	8 87	8 92	4 55	50 63	1 19	13 22	2 12	**25** 14	1 12	13 14
HLA-C Homozygosity Heterozygosity	8 87	8 92	5 54	63 62	3 17	**38** 20	2 12	**25** 14	1 12	13 14

Results analyzed in the main text are highlighted in bold.

**Table 6:** Genes affected by VARs associated with IRAE and organ specific IRAE. Note that we only list genes with observed incidence of IRAE above the respective cohort average and concerning at least two patients.

IRAE	Genes Affected by VARs	N of Patients with VARs and IRAE	% Patients with VARs Having IRAE
IRAE in general	*SMAD3* *PRDM1* *PDCD1* *IL1RN* *CD274*	8 29 3 31 3	67% 64% 100% 65% 75%
Colitis	*TERT* *SMAD3* *CTLA4* *IL1RN*	2 3 16 11	29% 25% 27% 23%
Pneumonitis	*PRDM1*	4	9%
Hepatitis	*PRDM1* *CTLA4*	7 10	16% 17%
Myocarditis	*SLCO1B1*	2	4%
Myositis	*IL1RN*	2	4%
Pancreatitis	*TERT* *SH2B3* *SMAD3* *FAN1* *IL1RN*	2 10 4 9 9	29% 15% **33%** (*p* = 0.034) 15% 19%
Exanthema	*PRDM1* *CTLA4*	4 5	9% 9%
Hypophysitis	*SMAD3* *PRDM1* *IL1RN*	3 6 7	25% 13% 15%
Nephritis	*FAN1* *IL1RN*	3 3	5% 6%
Thyroiditis	*CTLA4* *FAN1*	5 5	9% 8%

Results analyzed in the main text are highlighted in bold.

**Table 7:** Genes affected by CNVs (deletions or duplications) in relation to observed IRAE. Note that we only list genes with observed incidence of IRAE above the respective cohort average and concerning at least two patients.

IRAE	Gene Affected	Patients with CNV and IRAE		Deletions with IRAE		Duplications with IRAE	
		*N*	%	*N*	%	*N*	%
**IRAE**	*TERT* *LRRK2* *IKZF1* *SMAD3* *JAK2* *PRDM1* *CTLA4* *TSHR* *FAN1* *SLCO1B1* *PDCD1* *IL1RN* *CD274* *UNG*	31 24 27 25 28 24 10 16 29 39 19 13 14 22	69% 63% 68% 69% 65% 75% 71% 80% 73% 66% 68% **87%** (*p* = 0.033) 78% 71%	- 9 3 - 18 19 3 9 9 7 - 5 12 2	- 90% 75% - 78% **86%** (*p* = 0.026) 75% 90% 82% 64% - 100% 80% 67%	27 - 23 25 - - 6 - - 27 17 7 2 19	73% - 68% 69% - - 67% - - 66% 71% 78% 67% 70%
Colitis	*TERT* *IKZF1* *SMAD3* *FAN1* *IL1RN*	10 11 8 8 4	22% 28% 22% 20% 27%	2 - - 3 -	29% - - 27% -	- 11 8 - 3	- 32% 22% - 33%
Hepatitis	*SH2B3* *LRRK2* *IKZF1* *SMAD3* *PRDM1* *CTLA4* *TSHR* *FAN1* *SLCO1B1* *IL1RN* *CD274* *UNG*	5 8 7 8 5 3 5 8 13 4 4 5	19% 21% 18% 22% 16% 21% 25% 20% **22%** (*p* = 0.010) 27% 22% 16%	- 3 - - - - 2 - 2 - - -	- 30% - - - - 20% - 18% - - -	5 5 7 8 2 3 2 7 9 4 2 5	19% 19% 21% 22% 20% 33% 29% 28% 22% 44% **67%** (*p* = 0.043) 19%
Pancreatitis	*TERT* *IKZF1* *JAK2* *FAN1* *SLCO1B1* *IL1RN* *UNG*	5 5 4 4 7 2 3	11% 13% 9% 10% 12% 13% 10%	2 - - 2 - - -	29% - - 18% - - -	- 5 3 - 6 - -	- 15% 15% - 15% - -

Results analyzed in the main text are highlighted in bold.

**Table 8:** Genes affected by CNVs in general associated with IRAE. Note that we only list genes with observed incidence of IRAE above the respective cohort average and concerning at least two patients.

IRAE	Gene Affected by CNV	N of Patients with CNV and IRAE	% of Patients with CNV and IRAE
Pneumonitis	*PRDM1* *CD274*	3 2	9% 11%
Encephalitis	*TERT* *IKZF1* *JAK2* *PRDM1* *CD274*	3 2 3 3 2	7% 5% 7% **9%** (*p* = 0.014) **11%** (*p* = 0.032)
Myocarditis	*SLCO1B1*	2	3%
Myositis	*TERT* *LRRK2* *IKZF1* *SMAD3* *JAK2* *PRDM1* *TSHR* *FAN1* *CD274* *UNG*	2 2 2 2 2 3 2 3 2 2	4% 5% 5% 6% 5% **9%** (*p* = 0.014) **10%** (*p* = 0.049) **8%** (*p* = 0.039) **11%** (*p* = 0.032) 6%
Exanthema	*TERT* *SH2B3* *SMAD3* *JAK2* *PRDM1* *CTLA4* *TSHR* *SLCO1B1* *IL1RN* *CD274*	4 3 3 4 4 2 2 6 2 3	9% 11% 8% 9% 13% 14% 10% 10% 13% 17%
Hypophysitis	*TERT* *SMAD3* *JAK2* *PRDM1* *FAN1* *CD274* *UNG*	5 4 5 5 7 3 4	11% 11% 12% 16% 18% 17% 13%
Nephritis	*IKZF1*	2	5%
Thyroiditis	*LRRK2* *SMAD3* *PRDM1* *TSHR* *SLCO1B1* *PDCD1* *CD274* *UNG*	4 3 3 2 6 3 2 3	11% 8% 9% 10% 10% 11% 11% 10%

Results analyzed in the main text are highlighted in bold.

## Data Availability

The data presented in this study are available on request from the corresponding author. The data are not publicly available due to privacy.

## Supplementary Materials

The following are available online at https://www.mdpi.com/article/10.3390/cancers14020302/s1, Figure S1: Forest plots HLA homo- and heterozygosity depicting the confidence intervals and odds ratios on a logarithmic scale, Figure S2: Forest plots CNVs depicting confidence intervals and odds ratios on a logarithmic scale, Table S1: Sex-specific thresholds of the differential blood count according to the laboratory of the University Hospital Tübingen, Table S2: Alphabetical list of genes included in the two gene panels used for NGS by CeGat GmbH.
