# Supplementary material for: Biomarkers Associated with Immune-Related Adverse Events under Checkpoint Inhibitors in Metastatic Melanoma
Source: Cancers (Basel). 2022 Jan 8;14(2):302. doi: 10.3390/cancers14020302 (PMC8773840; doi:10.3390/cancers14020302)
Supplement: Supplementary file 1 [file cancers-14-00302-s001.zip › cancers-1537689-supplementary.pdf]

cancers

Supplementary Material: Biomarkers Associated with Immune-Related Adverse Events under Checkpoint Inhibitors in Metastatic Melanoma

Marcus Wölffer, Florian Battke, Martin Schulze, Magdalena Feldhahn, Lukas Flatz, Peter Martus and Andrea Forschner

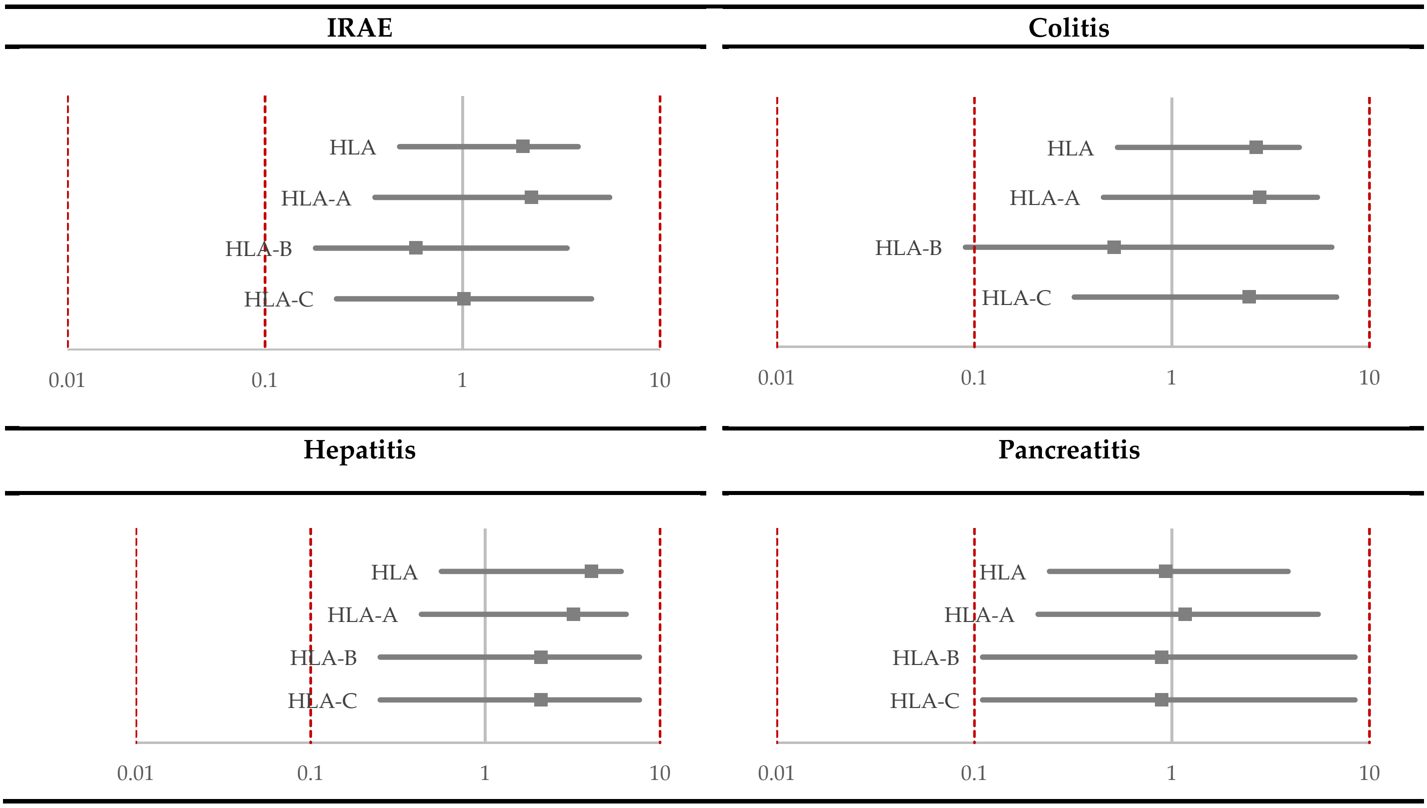

Figure S1. Forest plots HLA homo- and heterozygosity depicting the confidence intervals and odds ratios on a logarithmic scale. Note that all confidence intervals include 1, meaning that we cannot conclude that there is a statistically significant difference.

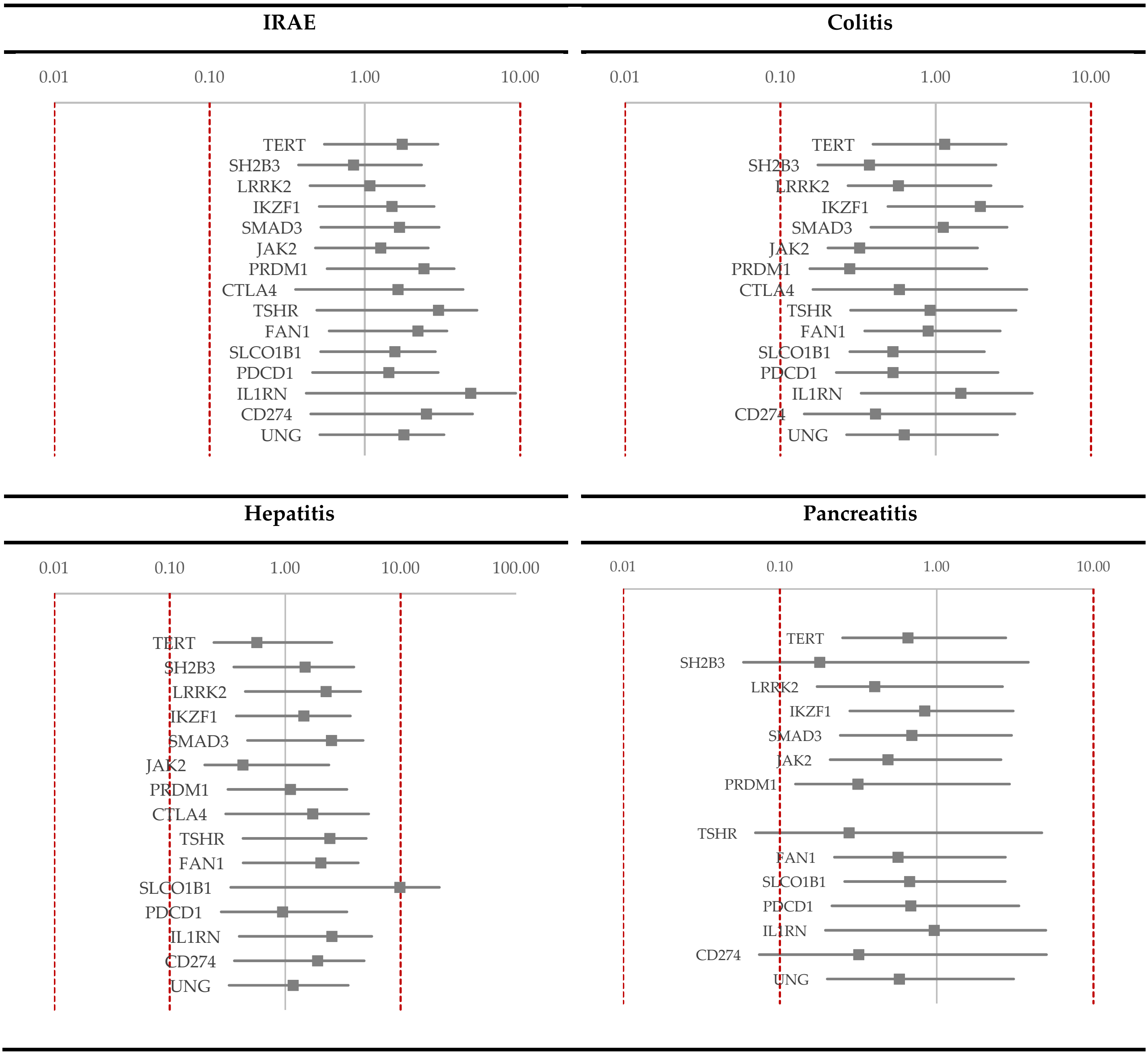

Figure S2. Forest plots CNVs depicting confidence intervals and odds ratios on a logarithmic scale. Note that all confidence intervals include 1, meaning that we cannot conclude that there is a statistically significant difference.

Table S1. Sex-specific thresholds of the differential blood count according to the laboratory of the University Hospital Tübingen.

Differential Blood Count	Thresholds Female Sex	Thresholds Male Sex
Leucocytes	4100–11800/µL	3800–10300/µL
Neutrophils Absolute	2100–7700/µL	1800–7000/µL
%	40–80%	40–80%
Lymphocytes Absolute	1200–3500/µL	1100–3200/µL
%	20–45%	20–45%
Monocytes Absolute	250–850/µL	260–870/µL
%	2–13%	2–13%
Eosinophils Absolute	30–470/µL	30–470/µL
%	0.5–8%	0.5–8%

Table S2. Alphabetical list of genes included in the two gene panels used for NGS by CeGat GmbH.

TUM.4, including 711 genes	TUM.5, including 742 genes
ABL1, ABL2, ACD, AIP, AJUBA, AKT1, AKT2, AKT3, ALK, AMER1, ANKRD26, APC, AR, ARAF, ARHGAP35, ARID1A, ARID1B, ARID2, ARID5B, ASXL1, ASXL2, ATG2B, ATM, ATP1A1, ATR, ATRX, AURKA, AURKB, AURKC, AXIN1, AXIN2, AXL, B2M, BAP1, BARD1, BCL10, BCL11A, BCL11B, BCL2, BCL3, BCL6, BCL9, BCOR, BCORL1, BCR, BIRC2, BIRC3, BIRC5, BLM, BMPR1A, BRAF, BRCA1, BRCA2, BRD3, BRD4, BRIP1, BTK, BTNL2, BUB1B, C11ORF30, CALR, CAMK2G, CARD11, CASP8, CBFB, CBL, CBLB, CBLC, CCDC6, CCND1, CCND2, CCND3, CCNE1, CD274, CD38, CD52, CD58, CD79A, CD79B, CD82, CDC73, CDH1, CDH11, CDH2, CDK12, CDK4, CDK6, CDK8, CDKN1A, CDKN1B, CDKN1C, CDKN2A, CDKN2B, CDKN2C, CEBPA, CEP57, CHD1, CHD2, CHD4, CHEK1, CHEK2, CIC, CIITA, CKS1B, CNOT3, COL1A1, COMMD1, CREB1, CREBBP, CRKL, CRTC1, CRTC2, CSF1R, CSF2, CSF3R, CSMD1, CSNK1A1, CTCF, CTLA4, CTNNA1, CTNNB1, CUL4B, CUX1, CXCR4, CYLD, CYP2A7, DAXX, DCC, DDB2, DDR1, DDR2, DDX11, DDX3X, DDX41, DEK, DHFR, DICER1, DIS3, DIS3L2, DKC1, DNMT1, DNMT3A, DOT1L, DPYD, EBP, EGFR, EGLN1, EGR2, EGR3, ELAC2, ELANE, ELF3, EML4, EP300, EPAS1, EPCAM, EPHA2, EPHA3, EPHA4, EPHB4, EPHB6, ERBB2, ERBB3, ERBB4, ERCC1, ERCC2, ERCC3, ERCC4, ERCC5, ERG, ERRFI1, ESR1, ESR2, ETNK1, ETS1, ETV1, ETV4, ETV5, ETV6, EWSR1, EXO1, EXT1, EXT2, EZH1, EZH2, FAM175A, FAM46C, FAN1, FANCA, FANCB, FANCC, FANCD2, FANCE, FANCF, FANCG, FANCI, FANCL, FANCM, FAS, FAT1, FBXW7, FES, FGF10, FGF14, FGF19, FGF2, FGF23, FGF3, FGF4, FGF5, FGF6, FGFBP1, FGFR1, FGFR2, FGFR3, FGFR4, FH, FKBP1A, FLCN, FLI1, FLT1, FLT3, FLT4, FOXA1, FOXA2, FOXE1, FOXL2, FOXO1, FOXO3, FOXP1, FOXQ1, FRK, FRS2, FUBP1, FUS, FYN, G6PD, GABRA6, GALNT12, GATA1, GATA2, GATA3, GATA4, GATA6, GLDN, GLI1, GLI2, GNA11, GNA13, GNAQ, GNAS, GPC3, GPER1, GPR124, GREM1, GRIN2A, GRM3, GSK3A, H3F3A, HCK, HGF, HIF1A, HIST1H3B, HLA-A, HLA-B, HLA-C, HLA-DPA1, HLA-DPB1, HLA-DQA1, HLA-DQB1, HLA-DRA, HLA-DRB1, HLF, HMGA2, HMGN1, HMOX2, HNF1A, HNF1B, HOXB13, HOXD8, HRAS, HSD3B1, HSP90AA1, HSP90AB1, ID3, IDH1, IDH2, IFNGR1, IFNGR2, IGF1R, IGF2, IGF2R, IKBKB, IKBKE, IKZF1, IKZF3, IL1B, IL1RN, IL2, IL21R, IL6, IL6ST, IL7R, ING4, INPP4B, INPPL1, IRF1, IRS2, ITK, JAK1, JAK2, JAK3, JUN, KAT6A, KDM5A, KDM5C, KDM6A, KDR, KEAP1, KIAA1549, KIT, KLF2, KLF4, KLHDC8B, KLHL6, KMT2A, KMT2B, KMT2C, KMT2D, KRAS, LATS1, LATS2, LCK, LIG4, LIMK2, LMO1, LRP1B, LRRK2, LTK, LYN, LZTR1, MAD2L2, MAFB, MAGEA1, MAGI1, MAGI2, MAML1, MAP2K1, MAP2K2, MAP2K3, MAP2K4, MAP2K5, MAP2K6, MAP2K7, MAP3K1, MAP3K14, MAP3K3, MAP3K4, MAP3K6, MAPK1, MAPK11, MAPK12, MAPK3, MAPK8IP1, MAX, MBD1, MC1R, MCL1, MDC1, MDM2, MDM4, MECOM, MED12, MEF2B, MEN1, MET, MGA, MGMT, MITF, MLH1, MLH3, MLLT10, MLLT3, MN1, MPL, MRE11A, MS4A1, MSH2, MSH3, MSH4, MSH5, MSH6, MSR1, MST1R, MTHFR, MTOR, MTRR, MUC1, MUC16, MUTYH, MXI1, MYB, MYC, MYCL, MYCN, MYD88, MYH11, MYH9, NBN, NCOA1,	ABL1, ABL2, ACD, ACVR1, AIP, AIRE, AJUBA, AKT1, AKT2, AKT3, ALK, AMER1, ANKRD26, APC, APLNR, AR, ARAF, ARHGAP35, ARID1A, ARID1B, ARID2, ARID5B, ASXL1, ASXL2, ATG2B, ATM, ATP1A1, ATR, ATRX, AURKA, AURKB, AURKC, AXIN1, AXIN2, AXL, B2M, BAP1, BARD1, BCL10, BCL11A, BCL11B, BCL2, BCL3, BCL6, BCL9, BCOR, BCORL1, BCR, BIRC2, BIRC3, BIRC5, BLM, BMPR1A, BRAF, BRCA1, BRCA2, BRD3, BRD4, BRIP1, BTK, BTNL2, BUB1B, C11ORF30, CALR, CAMK2G, CANX, CARD11, CASP8, CBFB, CBL, CBLB, CBLC, CCDC6, CCND1, CCND2, CCND3, CCNE1, CD274, CD38, CD52, CD58, CD74, CD79A, CD79B, CD82, CDC73, CDH1, CDH11, CDH2, CDK12, CDK4, CDK6, CDK8, CDKN1A, CDKN1B, CDKN1C, CDKN2A, CDKN2B, CDKN2C, CEBPA, CEP57, CHD1, CHD2, CHD4, CHEK1, CHEK2, CIC, CIITA, CKS1B, CNOT3, COL1A1, COMMD1, CREB1, CREBBP, CRKL, CRTC1, CRTC2, CSF1R, CSF2, CSF3R, CSMD1, CSNK1A1, CTCF, CTLA4, CTNNA1, CTNNB1, CTSB, CTSL, CTSS, CUL4B, CUX1, CXCR4, CYLD, CYP2A7, DAXX, DCC, DDB2, DDR1, DDR2, DDX11, DDX3X, DDX41, DEK, DHFR, DICER1, DIS3, DIS3L2, DKC1, DNMT1, DNMT3A, DOT1L, DPYD, E2F3, EBP, EGFR, EGLN1, EGR2, EGR3, ELAC2, ELANE, ELF3, EML4, EP300, EPAS1, EP-CAM, EPHA2, EPHA3, EPHA4, EPHB4, EPHB6, ERBB2, ERBB3, ERBB4, ERCC1, ERCC2, ERCC3, ERCC4, ERCC5, ERG, ERRFI1, ESR1, ESR2, ETNK1, ETS1, ETV1, ETV4, ETV5, ETV6, EWSR1, EXO1, EXT1, EXT2, EZH1, EZH2, FAM175A, FAM46C, FAN1, FANCA, FANCB, FANCC, FANCD2, FANCE, FANCF, FANCG, FANCI, FANCL, FANCM, FAS, FAT1, FBXW7, FES, FGF10, FGF14, FGF19, FGF2, FGF23, FGF3, FGF4, FGF5, FGF6, FGFBP1, FGFR1, FGFR2, FGFR3, FGFR4, FH, FKBP1A, FLCN, FLI1, FLT1, FLT3, FLT4, FOXA1, FOXA2, FOXE1, FOXL2, FOXO1, FOXO3, FOXP1, FOXQ1, FRK, FRS2, FUBP1, FUS, FYN, G6PD, GABRA6, GALNT12, GATA1, GATA2, GATA3, GATA4, GATA6, GLDN, GLI1, GLI2, GNA11, GNA13, GNAQ, GNAS, GPC3, GPER1, GPR124, GREM1, GRIN2A, GRM3, GSK3A, H3F3A, HCK, HGF, HIF1A, HIST1H3B, HLA-A, HLA-B, HLA-C, HLA-DPA1, HLA-DPB1, HLA-DQA1, HLA-DQB1, HLA-DRA, HLA-DRB1, HLF, HMGA2, HMGN1, HMOX2, HNF1A, HNF1B, HOXB13, HOXD8, HRAS, HSD3B1, HSP90AA1, HSP90AB1, HSPA4, ID3, IDH1, IDH2, IFI30, IFNGR1, IFNGR2, IGF1R, IGF2, IGF2R, IKBKB, IKBKE, IKZF1, IKZF3, IL1B, IL1RN, IL2, IL21R, IL6, IL6ST, IL7R, IL8, ING4, INPP4B, INPPL1, IRF1, IRS2, ITK, JAK1, JAK2, JAK3, JUN, KAT6A, KDM5A, KDM5C, KDM6A, KDR, KEAP1, KIAA1549, KIT, KLF2, KLF4, KLHDC8B, KLHL6, KMT2A, KMT2B, KMT2C, KMT2D, KRAS, LATS1, LATS2, LCK, LGMN, LIG4, LIMK2, LMO1, LRP1B, LRRK2, LTK, LYN, LZTR1, MAD2L2, MAFB, MAGEA1, MAGI1, MAGI2, MAML1, MAP2K1, MAP2K2, MAP2K3, MAP2K4, MAP2K5, MAP2K6, MAP2K7, MAP3K1, MAP3K14, MAP3K3, MAP3K4, MAP3K6, MAPK1, MAPK11, MAPK12, MAPK14, MAPK3, MAPK8IP1, MAX, MBD1, MC1R, MCL1, MDC1, MDM2, MDM4, MECOM, MED12, MEF2B, MEN1, MET, MGA, MGMT, MITF, MLH1, MLH3, MLLT10, MLLT3, MN1, MPL, MRE11A, MS4A1, MSH2, MSH3, MSH4, MSH5, MSH6, MSR1, MST1R, MTHFR, MTOR, MTRR, MUC1, MUTYH, MXI1, MYB, MYC, MYCL, MYCN,

NCOA3, NCOR1, NF1, NF2, NFE2L2, NFKB1, NFKB2, NFKBIA, NFKBIE, NIN, NLRC5, NOP10, NOTCH1, NOTCH2, NOTCH3, NOTCH4, NPM1, NQO1, NR1I3, NRAS, NRG2, NSD1, NT5C2, NTHL1, NTRK1, NTRK2, NTRK3, NUMA1, NUP98, PAK3, PALB2, PALLD, PARK2, PARP1, PARP2, PARP4, PAX3, PAX5, PAX7, PBK, PBRM1, PBX1, PDCD1, PDCD1LG2, PDF, PDGFA, PDGFB, PDGFC, PDGFD, PDGFRA, PDGFRB, PDK1, PGR, PHF6, PHOX2B, PIAS4, PIGA, PIK3C2A, PIK3C2B, PIK3C2G, PIK3CA, PIK3CB, PIK3CD, PIK3CG, PIK3R1, PIK3R2, PIK3R3, PIM1, PKHD1, PLCG1, PLCG2, PML, PMS1, PMS2, POLD1, POLE, POLH, POLQ, POT1, PPM1D, PRDM1, PRDM16, PREX2, PRF1, PRKAR1A, PRKCA, PRKD1, PRKDC, PROM2, PRSS1, PRX, PSIP1, PSMB1, PSMB10, PSMB2, PSMB5, PSMB8, PSMB9, PSMC3IP, PSPH, PTCH1, PTCH2, PTEN, PTGS2, PTK2, PTK7, PTPN11, PTPRC, PTPRD, PTPRT, RAC1, RAC2, RAD21, RAD50, RAD51, RAD51B, RAD51C, RAD51D, RAD54B, RAD54L, RAF1, RALGDS, RARA, RARB, RARG, RASA1, RASAL1, RB1, RBM10, RECQL4, REL, RET, RFC2, RFX5, RHBDF2, RHEB, RHOA, RICTOR, RINT1, RIPK1, RIT1, RNASEL, RNF2, RNF43, ROS1, RPL22, RPS20, RPS6KB1, RPTOR, RSF1, RUNX1, RYR1, SACS, SAMHD1, SAV1, SBDS, SCG5, SDHA, SDHAF2, SDHB, SDHC, SDHD, SEC23B, SEMA4A, SETBP1, SETD2, SETDB1, SF3B1, SGK1, SH2B1, SH2B3, SH2D1A, SHFM1, SHH, SIK2, SIN3A, SIRT1, SKP2, SLC26A3, SLIT2, SLX4, SMAD3, SMAD4, SMARCA4, SMARCB1, SMARCE1, SMC1A, SMC3, SMO, SOCS1, SOX11, SOX2, SOX9, SPEN, SPINK1, SPOP, SPRED1, SPTA1, SRC, SRD5A2, SRGAP1, SRP72, SRSF2, SSTR1, SSTR2, SSTR3, SSTR5, SSX1, STAG1, STAG2, STAT1, STAT3, STAT5A, STAT5B, STK11, SUFU, SUZ12, SYK, TAF1, TAF15, TAP1, TAP2, TBK1, TBL1XR1, TBX3, TCF3, TCF7L2, TCL1A, TEK, TERC, TERF2IP, TERT, TET1, TET2, TFE3, TGFBR2, TLR4, TLX1, TMEM127, TNF, TNFAIP3, TNFRSF11A, TNFRSF13B, TNFRSF14, TNFRSF1A, TNFRSF1B, TNFRSF25, TNFRSF8, TNFSF11, TNK2, TOP1, TOP2A, TP53, TP53BP1, TPX2, TRAF2, TRAF3, TRAF5, TRAF6, TRAF7, TRRAP, TSC1, TSC2, TSHR, TUBA4A, TUBB, TYMS, U2AF1, UBE2T, UBR5, UGT2B15, UGT2B7, UIMC1, UNG, USP34, USP9X, VEGFA, VEGFB, VHL, VKORC1, WAS, WASF3, WHSC1, WISP3, WRN, WT1, XIAP, XPA, XPC, XPO1, XRCC1, XRCC2, XRCC3, XRCC5, XRCC6, YAP1, ZFHX3, ZHX3, ZNF217, ZNRF3, ZRSR2	MYD88, MYH11, MYH9, NBN, NCOA1, NCOA3, NCOR1, NF1, NF2, NFE2L2, NFKB1, NFKB2, NFKBIA, NFKBIE, NFYA, NFYB, NFYC, NIN, NLRC5, NOP10, NOTCH1, NOTCH2, NOTCH3, NOTCH4, NPM1, NQO1, NR1I3, NRAS, NRG2, NSD1, NT5C2, NT5E, NTHL1, NTRK1, NTRK2, NTRK3, NUMA1, NUP98, PAK1, PAK3, PALB2, PALLD, PARK2, PARP1, PARP2, PARP4, PAX3, PAX5, PAX7, PBK, PBRM1, PBX1, PDCD1, PDCD1LG2, PDF, PDGFA, PDGFB, PDGFC, PDGFD, PDGFRA, PDGFRB, PDIA3, PDK1, PGR, PHF6, PHOX2B, PIAS4, PIGA, PIK3C2A, PIK3C2B, PIK3C2G, PIK3CA, PIK3CB, PIK3CD, PIK3CG, PIK3R1, PIK3R2, PIK3R3, PIM1, PKHD1, PLCG1, PLCG2, PML, PMS1, PMS2, POLD1, POLE, POLH, POLQ, POT1, PPM1D, PPP2R2A, PRDM1, PRDM16, PREX2, PRF1, PRKAR1A, PRKCA, PRKD1, PRKDC, PROM2, PRSS1, PRX, PSIP1, PSMB1, PSMB10, PSMB2, PSMB5, PSMB8, PSMB9, PSMC3IP, PSME1, PSME2, PSME3, PSPH, PTCH1, PTCH2, PTEN, PTGS2, PTK2, PTK7, PTPN11, PTPN12, PTPRC, PTPRD, PTPRT, RAC1, RAC2, RAD21, RAD50, RAD51, RAD51B, RAD51C, RAD51D, RAD54B, RAD54L, RAF1, RALGDS, RARA, RARB, RARG, RASA1, RASAL1, RB1, RBM10, RECQL4, REL, RET, RFC2, RFX5, RFXANK, RFXAP, RHBDF2, RHEB, RHOA, RICTOR, RINT1, RIPK1, RIT1, RNASEL, RNF2, RNF43, ROS1, RPL22, RPS20, RPS6KB1, RPTOR, RSF1, RUNX1, RYR1, SACS, SAMHD1, SAV1, SBDS, SCG5, SDHA, SDHAF2, SDHB, SDHC, SDHD, SEC23B, SEMA4A, SETBP1, SETD2, SETDB1, SF3B1, SGK1, SH2B1, SH2B3, SH2D1A, SHFM1, SHH, SIK2, SIN3A, SIRT1, SKP2, SLC26A3, SLIT2, SLX4, SMAD3, SMAD4, SMARCA4, SMARCB1, SMARCE1, SMC1A, SMC3, SMO, SOCS1, SOX11, SOX2, SOX9, SPEN, SPINK1, SPOP, SPRED1, SPTA1, SRC, SRD5A2, SRGAP1, SRP72, SRSF2, SSTR1, SSTR2, SSTR3, SSTR5, SSX1, STAG1, STAG2, STAT1, STAT3, STAT5A, STAT5B, STK11, SUFU, SUZ12, SYK, TAF1, TAF15, TAP1, TAP2, TAPBP, TBK1, TBL1XR1, TBX3, TCF3, TCF4, TCL1A, TEK, TERC, TERF2IP, TERT, TET1, TET2, TFE3, TGFB1, TGFBR2, TLR4, TLX1, TMEM127, TNF, TNFAIP3, TNFRSF11A, TNFRSF13B, TNFRSF14, TNFRSF1A, TNFRSF1B, TNFRSF25, TNFRSF8, TNFSF11, TNK2, TOP1, TOP2A, TP53, TP53BP1, TP63, TPX2, TRAF2, TRAF3, TRAF5, TRAF6, TRAF7, TRRAP, TSC1, TSC2, TSHR, TTK, TUBA4A, TUBB, TYMS, U2AF1, UBE2T, UBR5, UGT2B15, UGT2B7, UIMC1, UNG, USP34, USP9X, VEGFA, VEGFB, VHL, VKORC1, WAS, WASF3, WHSC1, WISP3, WRN, WT1, XIAP, XPA, XPC, XPO1, XRCC1, XRCC2, XRCC3, XRCC5, XRCC6, YAP1, YES1, ZFHX3, ZHX3, ZNF217, ZNRF3, ZRSR2
